# Sustainable synthesis of molecularly imprinted polymers using leuco fuchsin for selective detection of formaldehyde in food

**DOI:** 10.1039/d6ra02680j

**Published:** 2026-07-08

**Authors:** Meyliana Wulandari, Muhammad Al Faqih Fiddin, Dieta Putri Ayuserani, Rosi Fitri Ramadani, Syabina Khaila Aliffa, Nofrizal, Andreas, Pandian Bothi Raja, Thomas Nesakumar Jebakumar Immanuel Edison

**Affiliations:** a Department of Chemistry, Faculty of Mathematics and Natural Science, Universitas Negeri Jakarta 13220 Indonesia; b Department of Biology, Faculty of Mathematics and Natural Science, Universitas Negeri Jakarta 13220 Indonesia; c Department of Chemistry, Faculty of Science and Technology, State Islamic University (UIN) Syarif Hidyatullah Jakarta Ciputat Banten 15412 Indonesia; d Ministry of Energy and Mineral Resources – Lemigas Jl. Ciledug Raya Kavling. 109 Jakarta 12230 Indonesia; e Badan Riset dan Inovasi Nasional South Tangerang 15314 Indonesia; f School of Chemical Sciences, Universiti Sains Malaysia, Gelugor, Penang 11800 Malaysia; g Sethu Research and Innovation Centre (SRIC), Department of Chemistry, Sethu Institute of Technology Kariapatti 626115 Tamil Nadu India

## Abstract

Formalin (aqueous formaldehyde) is a well-documented human carcinogen often used as an illegal food preservative. The World Health Organization (WHO) limits the daily intake of formalin to 1.5–14 mg for adults. Higher dosages of formalin can cause death by throat swelling or lung burning. Hence, the need for an instant, selective, and sensitive method for detecting formalin is clear. The present work discusses the efficient synthesis of silica-supported molecularly imprinted polymers (SiO_2_@MIPs) for the selective and sensitive detection of formalin using a UV-visible spectrophotometer. This method offers rapid detection with high specificity and sensitivity, enabling timely detection of formalin in food matrices. The synthesized SiO_2_@MIPs are characterized using Fourier transform infrared (FTIR) spectroscopy, scanning electron microscopy with energy-dispersive X-ray spectroscopy (SEM-EDX), and a particle size analyzer (PSA). After reaction with SiO_2_@MIPs, formalin reacted colorimetrically with leuco fuchsin, resulting in a color change from pale yellow to purple. The adsorption parameters, including the effect of pH, time, and formalin concentration, were also investigated. The adsorption isotherms for formalin in the MIPs and the non-imprinted polymer (NIP) were fitted to the Freundlich isotherm model, and the calculated average binding affinities for formalin were 0.44 and 0.13 mg g^−1^ for the MIPs and NIP, respectively. Good recoveries and precision ranging between 98% and 105%, with relative standard deviation (RSD) of <5% (*n* = 3) in the range 0–10 mg L^−1^, were obtained. The Stöber method for silica coating is often considered more environmentally friendly. Iit effectively prevents structural damage to imprinting sites and ensures an adsorption capacity loss of <5% after 3 months, making the approach more sustainable. This method can serve as an alternative to formaldehyde detection in food samples, offering operational efficiency, enhanced selectivity, and environmental benefits.

## Abstract 2

Formalin (aqueous formaldehyde) is a well-documented human carcinogen often used as an illegal food preservative.

## Graphical abstract

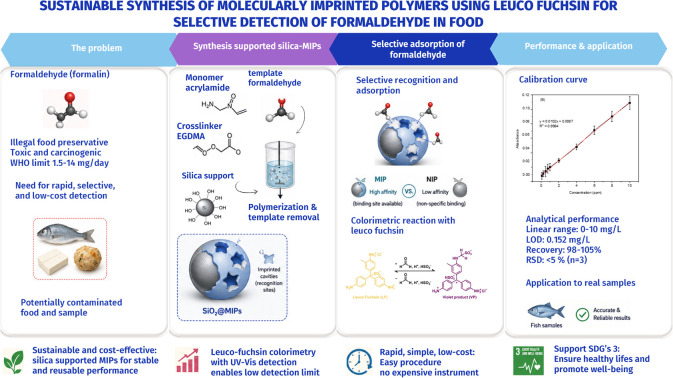

## Introduction

Formaldehyde is a colorless, reactive, and pungent compound. It is commonly used as a coating for paper and wood and as a preservative for corpses.^1^ Formaldehyde is still widely used as a food preservative, even though several international institutions have banned its use.^2^ The World Health Organization (WHO) limits oral exposure to formaldehyde to 1.5–14 mg per day for adults and 100 mg per day for short-term exposure.^3^ Bioaccumulation of formaldehyde in the body can cause inflammation of the liver and kidneys as well as irritation of the lungs' mucous membranes.^4^ Formaldehyde is a group 1 carcinogenic compound according to the International Agency for Research on Cancer (IARC).^5^ This observation underscores the necessity for sensitive detection methods capable of operating in the low mg L^−1^ to µg L^−1^ range.

Quantitative analysis of formaldehyde is generally performed using high-performance liquid chromatography (HPLC),^6^ gas chromatography,^7^ and gas chromatography coupled with mass spectrometry (GC-MS).^8^ However, not all laboratories have access to such expensive equipment. Hence, the use of rapid test kits is a common practice for formalin detection. These rapid test kits are less accurate because they can detect other aldehyde groups (false positives) and have a poor detection limit (0.5–2 mg L^−1^). Therefore, colorimetry and spectrophotometry remain preferred because they are more accurate, easier, and cheaper.^9,10^ However, the colorimetric method has low selectivity. Therefore, previous studies have reported the use of gold nanoparticles (AuNPs) for the colorimetric detection of formaldehyde, which provides high sensitivity but requires relatively complex fabrication procedures and instrumentation.^9^ Research using a quartz crystal microbalance (QCM) relies on changes in the quartz crystal's oscillation frequency upon binding target molecules. However, QCM sensors are less stable to environmental changes.^11,12^

The molecularly imprinted technology (MIT) detection method is a selective method designed to overcome some of these limitations. MIT consists of monomers that bind to a specific template molecule (the target) and are polymerized around it. Then, the template molecules are released from the polymer, leaving selective recognition sites for the target compound.^10^ The resulting polymers, called molecularly imprinted polymers (MIPs), exhibit high stability under various environmental conditions.^13^ MIPs are highly effective recognition materials due to their tailor-made selectivity, which complements target molecules in size, shape, and functionality. Unlike biological receptors, MIPs offer superior chemical and thermal stability, enabling reliable performance under harsh conditions. MIPs are cost-efficient, easy to synthesize, and feature a robust crosslinked structure that ensures reusability and a long shelf life. This versatility makes MIPs attractive artificial materials for a wide range of analytes, including small molecules such as formaldehyde, across sensing, separation, and sample preparation applications.^14–16^

Previous studies used molecular geometry simulations with the acrylamide monomer, achieving an optimal monomer-template ratio of 1 : 4, a MIP adsorption capacity of 54.67 mg g^−1^, a formaldehyde concentration of 400 mg L^−1^, and a contact time of 400 minutes.^17^ Other studies used methacrylic acid monomers, showing MIPs with good complex stability at a 1 : 3 monomer-template ratio.^18^ Electrochemically detected formaldehyde MIPs are highly selective but are complex, irreproducible, and less effective.^19^

The colorimetric method provides a fast, simple way to detect formaldehyde with MIPs, since it avoids the need for expensive instruments. For instance, previous studies used the colorimetric reagent fluoral-P, which produces 3,5-diacetyl-1,4-dihydrolutidine (DDL). However, the reaction between formaldehyde and fluoral-P remains unstable, often leading to detection errors.^20^ To address these limitations, Darder *et al.* (2022) developed a fiber-optic colorimetric sensor for formaldehyde based on leuco fuchsin, which changes color from pale yellow to purple upon reaction with formaldehyde.^21^ While leuco fuchsin directly detects formaldehyde in food, food matrices frequently interfere, compromising accuracy. To overcome these weaknesses, this study integrates MIPs with leuco fuchsin, thereby improving selectivity, specificity, and accuracy in formaldehyde detection. In addition, the synthesized SiO_2_@MIPs cost less than commercial kits and offer high stability due to the protective silica coating, allowing users to rely on them for routine analysis. Accordingly, this study aims to determine formaldehyde levels in food samples using MIPs–leuco fuchsin, providing a sustainable, reliable, and accessible approach to promoting well-being in food consumption (SDG 3).

MIPs were synthesized by the bulk method and then coated with silica using the Stöber method to prevent damage to the MIP active sites during grinding.^22^ Silica can increase the stability and biocompatibility of polymer structures,^23^ thereby increasing the adsorption capacity of MIPs. Unlike conventional MIPs that require instruments, this work pairs MIPs with a colorimetric system, enabling selective, straightforward visual detection. This combination bridges high selectivity (MIPs) with operational simplicity (colorimetry). The study includes characterization of the synthesized MIPs using Fourier transform infrared (FTIR) and scanning electron microscopy-energy dispersive X-ray (SEM-EDX) and analysis of the adsorption capacity of the MIPs as selective adsorbents for formaldehyde, in addition to determining the isotherm adsorption, selectivity, and stability of the material. Furthermore, validation was conducted to determine the performance of the resulting MIPs and to establish their suitability for applications in the analysis of food samples. In this study, the non-imprinted polymer (NIP) was also synthesised as a negative control, *i.e.*, without the addition of formaldehyde during the synthesis. The application to real food matrices, specifically fish samples, was reported.

## Experimental

### Materials

The following chemicals were purchased from Merck (Germany): Tetraethyl orthosilicate (TEOS), formaldehyde (37%), methanol (99%), ethanol (99%), acetonitrile, glacial acetic acid, benzoic acid, hydrochloric acid, formic acid, and sodium hydroxide. Acrylamide 98%, 2,2′-azobis(isobutyronitrile) (AIBN), ethylene glycol dimethacrylate (EGDMA) 98%, and basic fuchsin were purchased from Sigma-Aldrich. Whatman 42 filter paper and Millipore water were used. The equipment used included glassware commonly found in chemistry labs, as well as a UV-vis spectrophotometer (Shimadzu 1800), a Fourier transform infrared (FTIR) spectrometer (Shimadzu Prestige-21), and a scanning electron microscope with energy-dispersive X-ray (SEM-EDX) capabilities (JEOL JSM-IT200).

### Synthesis of silica as a coating

This synthesis aims to improve the mechanical properties of the polymer. A total of 0.025 mol of TEOS in 14.57 mL of ethanol was mixed with 0.1 mol of ammonia and 4.5 mL of H_2_O. The mixture was stirred for 30 minutes and then dried in an oven at 80 °C for 6 hours. It was subsequently calcined at 120 °C for 24 hours.^24^

### MIPs and NIP polymerisation

This synthesis was carried out using a bulk polymerisation method (the ratios of template, monomer, and crosslinker were 1 : 4 : 20 mmol). MIPs were synthesised by dissolving 0.352 mmol of acrylamide monomer, 1.76 mmol of EGDMA, and 15 mL of acetonitrile. The mixture was stirred for 2 hours, then 500 mg of SiO_2_ was added, and the mixture was stirred for 24 hours. 0.088 mmol of formaldehyde and 100 mg of AIBN were added to the mixture and polymerized at 60 °C until the polymer formed as MIPs-formaldehyde. The resulting MIPs-formaldehyde were then sieved using an agate mortar. The resulting MIPs were washed with acetonitrile and ethanol, and then dried in an oven at 50 °C for 24 hours. Control polymers (NIP) were prepared in the same way without formaldehyde as a template.^25^

### Template molecule extraction

MIPs-formaldehyde was extracted using a 90 : 10 (v/v) methanol: acetic acid solution, and the solvent was changed every 1 hour.^26^ The MIPs were washed and filtered repeatedly (24 times) until the template was extracted, as monitored by UV-vis spectrophotometric analysis of the washed solution.

### Percentage yield and purity of the synthesized materials

The percentage yield of the synthesized MIPs and NIP was calculated gravimetrically using the following equations:
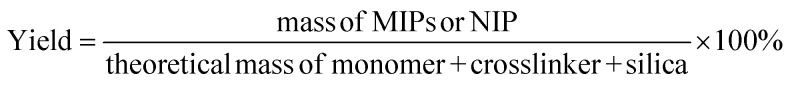
where the mass of MIPs and the NIP is the mass of the polymer after polymerization, washing, and drying at 50 °C for 24 h.

### Preparation of leuco fuchsin solution

A known weight of 0.05 grams of basic fuchsin was dissolved in 100 mL of distilled water, then 1.53 mL of NaHSO_3_ and 3.2 mL of phosphoric acid were added to the mixture while stirring.^27^

### Characterization of functional groups using FTIR, and the morphology of the MIPs and NIP using SEM-EDX

First, two grams of MIPs, template-free MIPs, and NIP were each mixed with 100 mg of KBr powder until a homogeneous mixture was achieved. Second, the resulting pellets were placed in a container and scanned in the range of 4000–500 cm^−1^.^28^ For the SEM measurements, the pellets were transferred to a sample container, methanol was dropped onto the surface, and an argon beam was fired to penetrate the surface; the signal was captured by the detector.^29^

### Absorption capacity of MIPs and the NIP on formaldehyde analysis

MIPs (30 mg) and the NIP were placed in 10 Erlenmeyer flasks containing 10 mL of a 3 mg L^−1^ formaldehyde solution at pH values ranging from 1 to 10. The MIPs and NIP were incubated for 30 minutes at 25 °C and 200 rpm, then filtered. The pH values of the collected formaldehyde solutions were adjusted to 4.65 ± 0.05 by slowly adding 0.1 M HCl or NaOH as needed. Next, two drops of 1% leuco fuchsin were added, and absorbance was measured at 549 nm using a UV-vis spectrophotometer. Optimum contact time and concentration were determined by varying the incubation duration (0–180 minutes) and formaldehyde concentrations (5–45 mg L^−1^). Adsorption capacity was calculated using the following equation:^30^
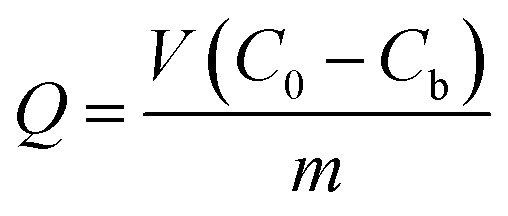
where *V* is the volume of solution used (L), *C*_0_ is the initial concentration of the formaldehyde solution (mg L^−1^), *C*_b_ is the concentration of the formaldehyde solution after contact (mg L^−1^), and *m* is the mass of MIPs and the NIP used (g).

### Measurement of formaldehyde concentration in food samples

Fresh fish samples were obtained from the East Jakarta market, cut into small pieces, the spines were removed, and the samples were crushed. A known weight (4 grams) of the fish sample was extracted with 50 mL of distilled water (pH 2, conditioned with 0.5 M HCl) using sonication for 30 minutes. The sample extract was then centrifuged (15 minutes at 10 000 rpm), and the supernatant was taken for further analysis. The rapid analysis method involved reacting 10 mL of the sample extract with 30 mg of MIPs for 10 minutes using a vortex mixer, followed by desorption for an additional 10 minutes. The desorption results were obtained, a 1 mL aliquot of the extract diluted to 50 mL with distilled water, and the pH was adjusted to 4.65 ± 0.05. Then, two drops of 1% leuco fuchsin were added, and the absorbance was measured at 549 nm.

## Results and discussion

### Synthesis of silica as the MIP coating

The Stöber method was chosen to produce mesoporous silica using tetraethyl orthosilicate (TEOS) as a precursor. The precursor undergoes hydrolysis and condensation, forming a colloidal silica sol, which then forms an ordered silica gel network.^31^ Ethoxy groups (Si–OR) in TEOS are replaced with silanol groups (Si–OH) *via* nucleophilic attack by OH^−^ ions.^32^ Ammonia acts as a base, generating OH^−^ ions, and as a catalyst, enhancing the reaction rate and interaction with silicon centers.^33^ During condensation, siloxane bonds (Si–O–Si) form as NH_3_ promotes deprotonation of silanol groups, which supports nucleophilic attack and the build-up of the silica network.^34^ Increased deprotonation from ammonia accelerates condensation.^35^ The Si/H_2_O molar ratio also affects the kinetics of hydrolysis and condensation. Residual reactants and by-products are removed by drying and calcination, improving the stability of the mesoporous silica framework.^36^

**Fig. 1:**
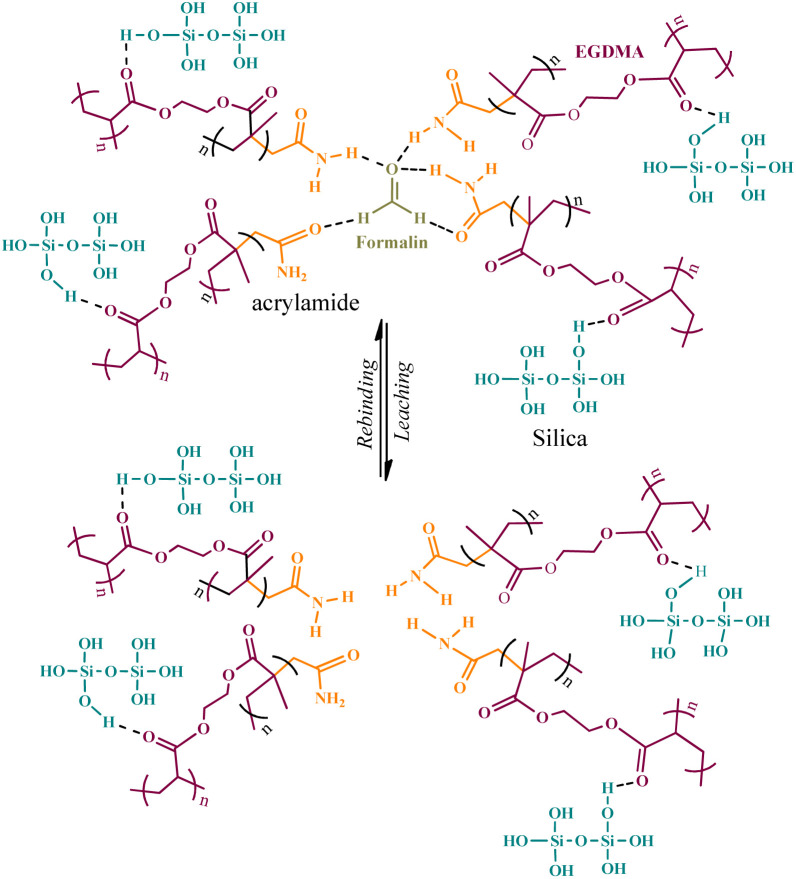
Possible polymerization mechanism between the template and the polymer backbone.

Fig. 1 shows that the molecular recognition mechanism of SiO_2_@MIPs for formaldehyde relies on a non-covalent imprinting strategy involving hydrogen bonding and dipole–dipole interactions. In the pre-polymerization stage, formaldehyde (the template) forms hydrogen bonds with the amide groups of the acrylamide monomers, forming a stable template–monomer complex.^37^ Upon initiation with AIBN, radical polymerization proceeds in the presence of the crosslinker EGDMA, producing a rigid three-dimensional polymer network that immobilizes the template within the matrix. Subsequent solvent extraction removes the template molecules, creating complementary binding cavities that retain the size, shape, and f-group orientation.^38^ The SiO_2_ coating, synthesized using the Stöber method, enhances structural stability and preserves the integrity of these imprinted cavities by protecting them from mechanical deformation and collapse. During the rebinding process, formaldehyde molecules are selectively recognized through the re-establishment of hydrogen bonding with the amide groups and additional dipole–dipole interactions within the imprinted sites. In contrast, the non-imprinted polymer (NIP) lacks these specific recognition sites and displays only non-specific adsorption. This distinction demonstrates that the selectivity of the SiO_2_@MIPs system results from the imprinting effect and the structural stability provided by the silica coating. Based on the calculations, it can be concluded that the obtained yield for MIPs before extraction is in the range of 90–92%. Meanwhile, the yield of the template-free MIPs is 80–85%, and the yield of the NIP is 90%. The % yield of the template-free MIPs is slightly lower than that of template-containing MIPs. This is likely due to the loss of a fraction of cross-links during template extraction.

### Template extraction from the MIPs and characterization by FTIR and SEM-EDX

The process of extracting formaldehyde templates from the MIPs involves the use of a solvent mixture of methanol and acetic acid (9 : 1 v/v). This solvent system is widely used in molecular imprinting because it efficiently disrupts non-covalent interactions while supporting the desorption mechanism, which depends on breaking hydrogen bonds and dipole–dipole interactions between formaldehyde and the functional monomer (acrylamide) within the imprinted polymer. Acetic acid acts as a proton donor, weakening and breaking the hydrogen bonds between the carbonyl group of formaldehyde and the amide groups of the polymer.^39^ Methanol, as a polar organic solvent, increases the solubility of the released formaldehyde and promotes its diffusion from the polymer matrix. These combined effects enable efficient template removal while preserving the structural integrity of the imprinted polymer.^40^

**Fig. 2:**
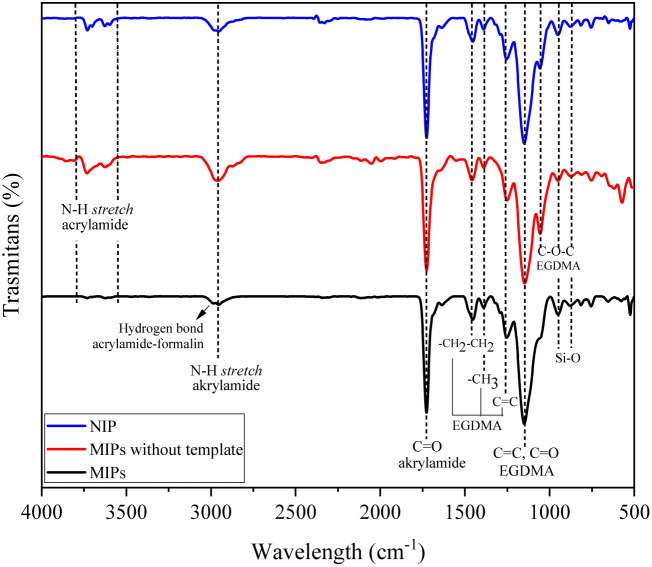
FTIR spectra of the template-MIPs, template-free MIPs, and NIP.

The FTIR spectra of templated MIPs and the NIP exhibit broadly similar features with distinct differences (Fig. 2). For the MIPs, an absorption band at 2984 cm^−1^ is attributed to hydrogen bond vibrations between formaldehyde and acrylamide. In contrast, the NIP displays bands at 3729 and 3627 cm^−1^, corresponding to N–H stretching vibrations of the acrylamide monomer. The absence of these bands in the MIPs indicates that the amine groups are involved in strong hydrogen bonding with formaldehyde, consistent with previous reports.^41^ Additional absorption bands at 1635–1636, 1452–1453, and 1390 cm^−1^ are assigned to C=C, CH_2_–CH_2_, and methyl groups of EGDMA, respectively. Furthermore, bands observed in the range of 877–950 cm^−1^ are characteristic of Si–O vibrations, confirming the incorporation of silica as a structural component in both MIPs and the NIP.

The efficiency of the template extraction was characterized by FTIR analysis. Successful template extraction is indicated by the disappearance of the hydrogen bond between formaldehyde and acrylamide, *i.e.*, the absorption band at 2984 cm^−1^. The appearance of absorptions at 3731 and 3626 cm^−1^ is consistent with the NIP spectra, indicating N–H stretching vibrations of acrylamide, following removal of the formaldehyde template. It can be concluded that, after the extraction of the template molecules, the spectra of MIPs and the NIP become identical.^42^

**Fig. 3:**
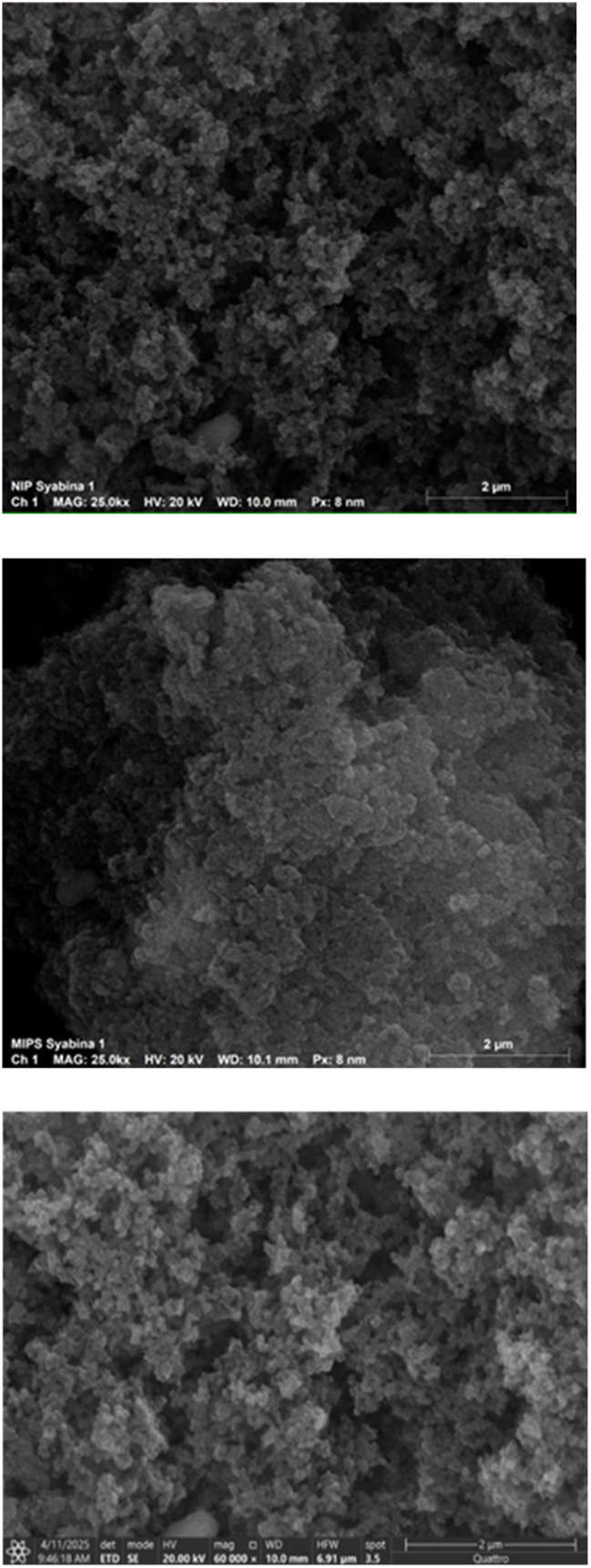
SEM images showing the surface morphology of NIPs (top), MIPs (middle), and MIPs after extraction (bottom).

**Fig. 4:**
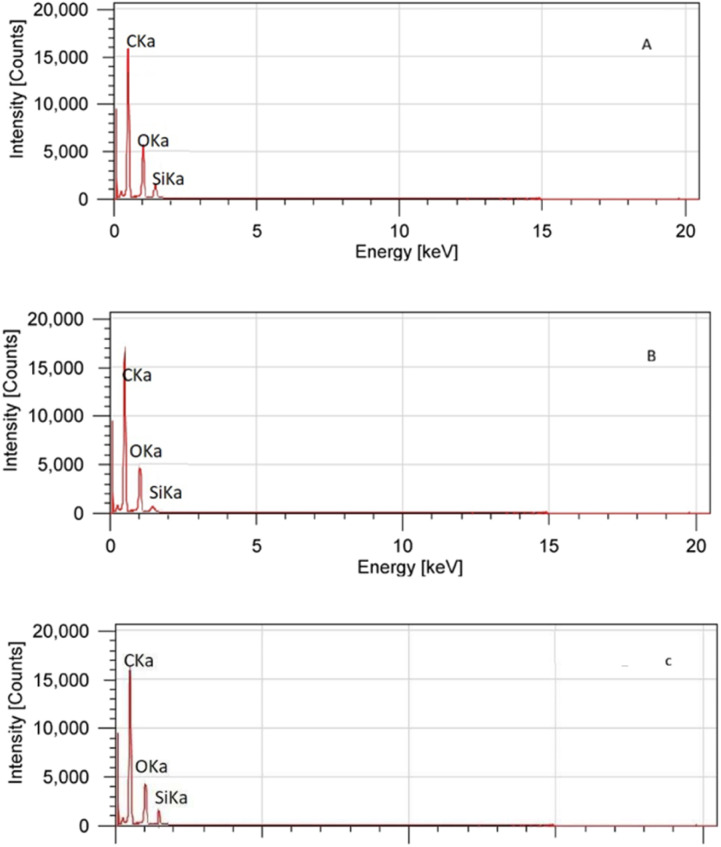
EDX spectra of the NIP (A), MIPs (B), and template-free MIPs (C).

**Fig. 5:**
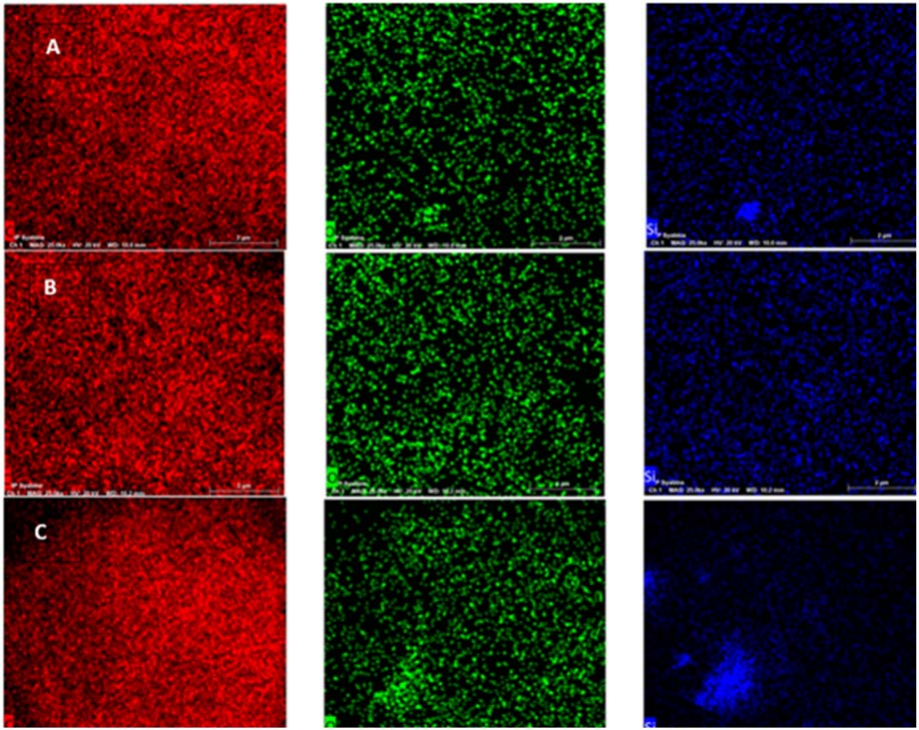
Elemental mapping of the NIP (A), MIPs (B), and template-free MIPs (C).

Fig. 3 shows the polymer morphology as determined by scanning electron microscope (SEM) characterization. The MIPs before template extraction showed a smooth, uniform surface. At the same time, the NIP displayed a hollow, porous surface. The presence of a template results in a relatively smooth, uniform surface. MIPs after template extraction produced obvious surface cavities. The SEM results were supported by EDX analysis (Table 1 and Fig. 4). The %C of MIP decreased after template extraction, approaching the %C of the NIP. This confirms the effectiveness of methanol and acetic acid as solvents for extracting formaldehyde. On the other hand, the %Si showed 0.32% for template-free MIPs and 0.17% for MIPs with a template. From these data, there was an increase in %Si after template extraction. The higher silicon content observed in the NIP (0.97%) suggests a more dominant silica contribution on the polymer surface, consistent with previous reports.^43^ The elemental mapping is depicted in Fig. 5.

**Table 1:** Elemental content in MIPs and NIP

Sample	Elemental content (% wt)		
	C	O	Si
NIP	89.41	9.62	0.97
MIPs	89.92	9.90	0.17
Template-free MIPs	87.53	12.15	0.32

As shown in Fig. 5, the mapping of silicon (blue) and oxygen (green) reveals a highly uniform, dense distribution across all three samples. The ubiquitous presence of Si confirms that the silica coating, synthesized *via* the Stöber method, successfully encapsulated the polymer core. The consistent density of silicon across the NIP, MIP, and template-free MIP suggests that the presence or absence of the formaldehyde template does not interfere with the formation of the rigid silica scaffold. The carbon mapping (red) represents the organic polymer backbone. In all images, carbon is distributed homogeneously, indicating a well-integrated composite where the organic molecularly imprinted polymer is evenly dispersed within or under the silica inorganic layer. This homogeneity is crucial to ensuring that the imprinted cavities are accessible across the entire surface of the material. Comparing the MIP (b) and template-free MIP (c), the elemental mapping maintains structural consistency, which indicates that the leaching process (template removal) is non-destructive to the SiO_2_ scaffold. While EDX mapping is primarily qualitative, the stability of the O and Si signals in sample (c) indicates that the functional framework remains intact after formaldehyde extraction. This structural integrity is essential for preserving the shape and size of the “tailor-made” cavities required for selective formaldehyde rebinding.

**Fig. 6:**
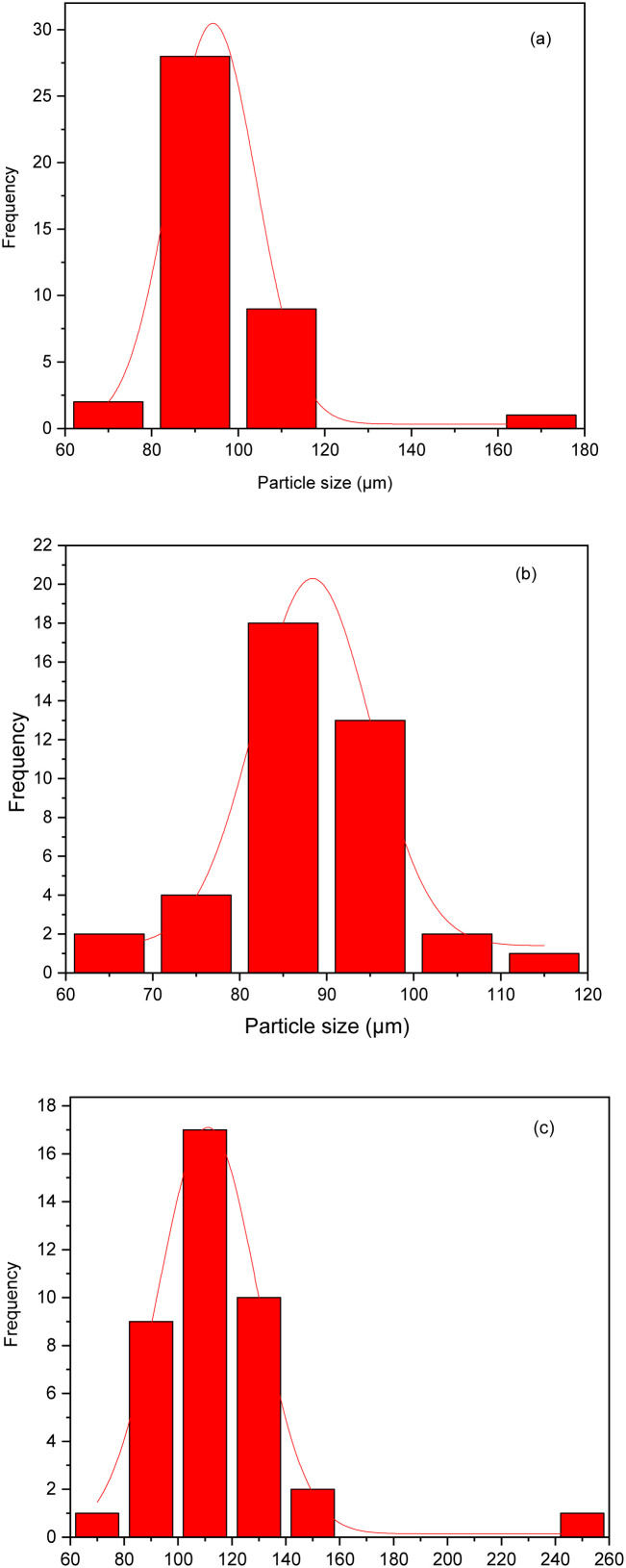
Particle size measurement of MIPs (a), template-free MIPs (b), and the NIP (c).

Particle size determination (Fig. 6) was performed using the ImageJ software (version 1.54 p) to evaluate the particle diameter distribution of the MIPs (a), template-free MIPs (b), and NIP (c). MIPs before extraction had a pore diameter of 94.15 µm (standard deviation 0.35, *R*^2^ 0.9988), template-free MIPs had a pore diameter of 88.73 µm (standard deviation 0.35, *R*^2^ 0.9978), and the NIP had a diameter of 111.06 µm (standard deviation 0.19, *R*^2^ 0.9964). The pore diameter data supported the SEM morphology. Template-free MIPs had a smaller mean diameter, reflecting the removal of entrapped formaldehyde and the formation of active sites.

The mean diameter of the NIP was larger due to its random morphology in the absence of a template. In this study, silica films were synthesized *via* the Stöber method without surfactant templating agents; therefore, the structure is expected to be irregular porous silica, not highly ordered mesoporous silica.^44^

### Synthesis of leuco fuchsin from basic fuchsin

Basic fuchsin is sulfonated with sodium bisulfite (NaHSO_3_) under acidic conditions to produce leuco fuchsin. During reduction, the SO_3_ group derived from NaHSO_3_ binds to the central carbon, saturating the conjugated system and yielding the colorless leuco fuchsin form. The % yield is calculated by dividing the mass of the dry synthesized leuco fuchsin by the mass of the basic fuchsin. The mass of basic fuchsin used is 0.005 g, and the resulting leuco fuchsin is 0.0043 g. Therefore, the % yield is 86%. The nucleophilic addition of bisulfite to the central quinoid carbon of triphenylmethane produces a colorless conjugated (leuco) chromophore with no side reactions. Leuco fuchsin is stable when stored at 4 °C for 30 days.

### Determination of the optimum conditions for the adsorption of MIPs and the NIP on formaldehyde

The study of analyte adsorption at the active sites of MIPs was conducted as a function of analyte pH. Fig. 6a emphasizes the increase in adsorption capacity from 1.17 to 1.22 mg g^−1^ from pH 1 to 2. This effect is influenced by increased electrostatic interactions between the protonated formaldehyde and the negatively charged polymer surface (due to the deprotonated silanol groups on the silica layer).^45^

Protonated analytes will be attracted by the negatively charged polymer surface, resulting in efficient adsorption.^46^ The adsorption capacity gradually decreases, reaching 0.63 mg g^−1^ at pH 8 due to decreased electrostatic interactions at higher pH. The adsorption capacity of the NIP has a similar trend to that of MIPs, but the value is lower.

Studies on diffusion kinetics, mass-transfer behavior, and analyte binding to the active sites of the MIPs were conducted over varying contact times. The adsorption capacity rose sharply within the first minute to 50 minutes, reaching 0.83 mg g^−1^ (MIPs) and 0.53 mg g^−1^ (NIP). As a result, 50 minutes was selected as the optimum adsorption time. After 50 minutes, only a slight increase in adsorption was noticed. Most of the active sites on the polymer appeared to be occupied by the analyte at this stage. Intraparticle diffusion and adsorption equilibrium govern this process. Such behavior aligns with previous reports that describe saturation of binding sites and partial desorption at prolonged contact times.^47^

**Fig. 7:**
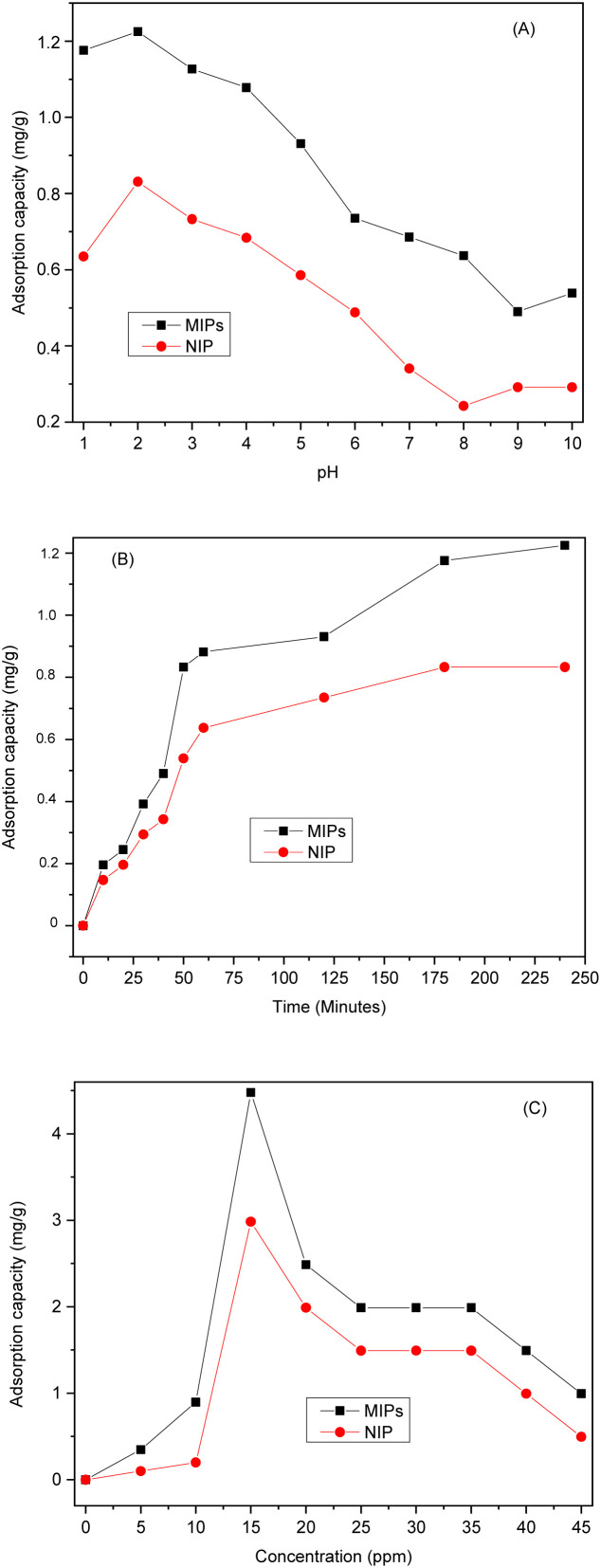
The effect of pH (a), contact time (b), and optimum concentration of MIPs and the NIP on the adsorption of target molecules (c).

The effect of initial analyte concentration on the adsorption performance was evaluated to determine the maximum uptake capacity for the MIPs and the NIP. Formaldehyde concentrations ranged from 5 to 45 ppm. Fig. 7c shows that the MIP adsorption capacity increased from 0.34 mg g^−1^ at 5 ppm to a maximum of 4.47 mg g^−1^ at 15 ppm, reflecting progressive occupation of imprinted binding sites. Beyond 15 ppm, the adsorption capacity declined, suggesting the adsorbent was saturated.^48^ Both MIPs and the NIP showed an optimal adsorption concentration of 15 mg L^−1^. However, MIPs showed significantly higher adsorption capacity (4.47 mg g^−1^) than the NIP (2.98 mg g^−1^), highlighting the enhanced binding affinity of molecular imprinting.

### Determination of binding site distribution and affinities

**Fig. 8:**
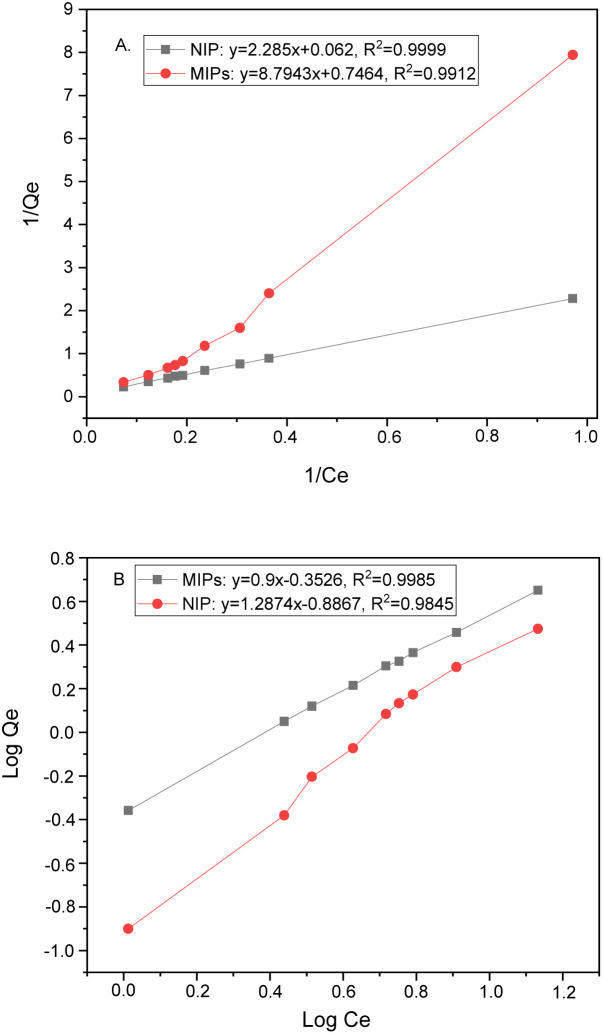
Equilibrium data for formalin uptake by MIPs and the NIP using the Langmuir (A) and the Freundlich (B) models.

Adsorption isotherms describe the amount of adsorbate retained on an adsorbent surface at a fixed temperature when the system reaches equilibrium. Equilibrium data were analyzed using the Langmuir and the Freundlich models. The following equation defines the logarithmic form of the Freundlich model.log *Q*_e_ = 1/*n* log *C*_e_ + log *K*_F_where *Q*_e_ is the amount of adsorbate at equilibrium (mg g^−1^), *C*_e_ is the equilibrium concentration of the adsorbate, *K*_F_ is the Freundlich constant related to adsorption capacity, and *n* is the constant related to the intensity of adsorption and is proportionate to the heterogeneity factor.^49^ The plots of log *C*_e_*vs.* log *Q*_e_ ought to yield a linear graph; the values of *n* and *K*_F_ can then be obtained from the slope and intercept of the graph, respectively (Fig. 8).

The *q*_max_ represents an equilibrium formalin concentration (mg g^−1^), while *K*_L_ relates to the adsorption capacity (mg g^−1^), which is the Langmuir constant. *K*_L_ is matched with the appropriate range variation and adsorbent porosity. The adsorption capacity is high due to the large surface area and pore volume. The formalin isothermal adsorption model on MIPs was statistically evaluated using the *R*^2^ values shown in Fig. 8A.
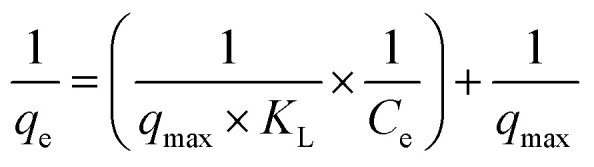


The linear regression of formalin yielded *R*^2^ values of 0.9999 (NIP) and 0.9912 (MIPs) using the Langmuir calculation model. The results show that the maximum adsorption capacity (*Q*_max_) was 16.12 mg g^−1^ (NIP) and 1.33 mg g^−1^ (MIPs), with Langmuir constants (*K*_L_) of 0.027 (NIP) and 0.085 (MIPs). This is apparently due to NIP's binding properties. MIPs have a limited number of specific binding sites. These sites match the number of template molecules removed. In contrast, NIPs lack these specific sites; instead, they use nonspecific adsorption on their surfaces. As a result, when analyte concentrations are high, NIPs adsorb more molecules at random. MIPs, however, bind analytes only within their unique cavities. Therefore, the adsorption isotherms fit the Freundlich (Fig. 8B) model well, with high *R*^2^ values (0.9985 for MIPs, 0.9845 for NIPs). The Freundlich model describes adsorption on heterogeneous surfaces—those with sites of varying adsorption energies. The constants *n* and *K*_F_ (where *n* describes how heterogeneous the surface is and KF indicates how strongly the material binds the adsorbate) were 1.11 and 0.44 mg g^−1^ for MIPs, and 0.13 mg g^−1^ and 0.77 for NIPs, respectively (Table 2). The *n* values show pronounced surface heterogeneity. The much higher *K*_F_ for the MIPs confirms their superior affinity and adsorption capacity for formaldehyde, compared to the NIP, highlighting the effectiveness of molecular imprinting for selective binding.

**Table 2:** Fitting of the adsorption isotherm

NIP	Langmuir	Freundlich
MIPs	*R* ^2^ = 0.9999	*R* ^2^ = 0.9945
	*Q*_max_ = 16.12	*K*_F_ = 0.13
	*K*_L_ = 0.027	*n* = 0.77
	*R* ^2^ = 0.9912	*R* ^2^ = 0.9985
	*Q*_max_ = 1.33	*K*_F_ = 0.44
	*K*_L_ = 0.085	*n* = 1.11

### Batch binding MIP selectivity test

Cross-selectivity was evaluated by contacting 30 mg of MIPs and the NIP with 3 mg L^−1^ solutions of formaldehyde, benzoic acid, and formic acid. After 6 h desorption, the eluates were evaporated, reconstituted in water, adjusted to pH 5, and reacted with 1% leuco fuchsin. Absorbance was measured at 549 nm. The imprinting factor was the ratio of the MIP adsorption capacity to the NIP adsorption capacity (Table 3). The results showed MIPs adsorbed more formaldehyde than the NIP under identical conditions, indicating the formation of specific recognition sites for the target molecule. NIP adsorption was mainly due to nonspecific interactions. Benzoic acid was chosen as a typical food preservative, and formic acid as a structural analogue. The MIPs were synthesized using formaldehyde as the template, which is the smallest aldehyde (CH_2_O). As a result, small-chain aldehydes are unlikely to be present in food matrices. Larger analogs, such as acetaldehyde or propionaldehyde, are too large to enter these cavities, which significantly reduces their potential for interference. The recognition mechanism relies not only on the presence of a carbonyl group but also on the spatial arrangement of functional groups, which correspond to the size and shape of formaldehyde. Table 3 describes the interference capacity factor, which exceeds that of the target analyte, which can be attributed to non-specific surface interactions. However, the interference factor was lower because the cavities did not accommodate the interfering species. This outcome strongly indicates the high quality of molecularly imprinted polymers (MIPs), demonstrating that MIPs function as selective sensors that can specifically recognise the target template in the presence of background interference.

**Table 3:** Evaluation of the imprinting factor of MIPs

Analyte	*k*_MIPs_	*k*_NIP_	IF^25^ (*k*_MIP_/*k*_NIP_)
Formaldehyde	0.44	0.13	3.38
Benzoic acid	1.20	1.38	0.87
Formic acid	1.18	1.57	0.75

### Determination of formaldehyde in fish samples

**Fig. 9:**
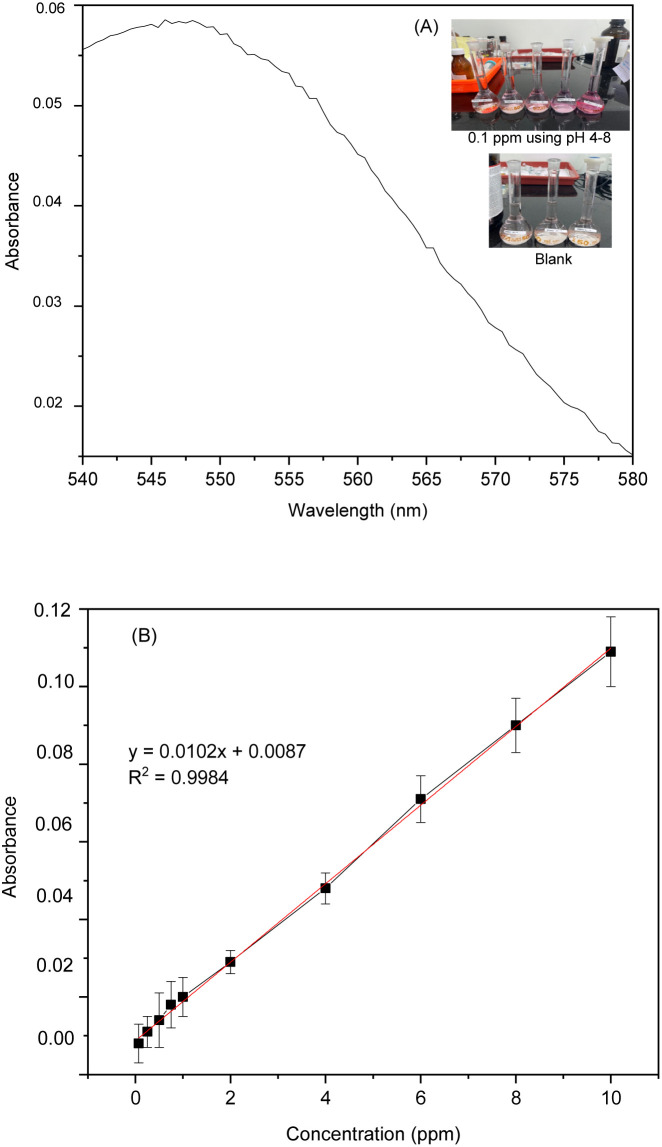
Maximum wavelength of leuco fuchsin (A) and the linearity curve of formaldehyde-leuco fuchsin (B).

An optimized protocol was used to analyze fish extracts containing formalin for method applicability. The accuracy of the MIPs method was assessed by studying the percentage recovery, indicating agreement between the measured and fortified concentrations at three levels (1–2 mg L^−1^; *n* = 3). Results are listed in Table 4. A linear calibration curve (0.1–10 mg L^−1^) was obtained by plotting standard concentration (*x*-axis) *versus* absorbance. The coefficient of determination (*R*^2^) was 0.9984, indicating excellent linearity.^50^ Maximal lambda and formalin linearity curves are shown in Fig. 9.

**Table 4:** Accuracy and precision of the MIPs-formalin method

Spiked (ppm)	Found concentration (ppm)	Recovery (%)	Precision (RSD, %)	
			Intra-day (*n* = 3)	Inter-day (*n* = 3)
0	0	—	—	—
1	1.2	102	4.8	3.2
1.5	1.57	104.6	4.2	4.0
2	1.97	98.5	3.5	3.1

Accuracy is calculated as (found concentration/spiked concentration) × 100%. Good accuracy is indicated by recovery between 98–105% (RSD 2.4–5%, *n* = 3). Repeatability (inter-assay precision) refers to the method's results over a short time under identical conditions.^51^ Data on the repeatability and reproducibility of peak areas were obtained by injecting a 2 ppm standard on the same day (intra-day) and over three consecutive days (inter-day). Repeatability was determined using %RSD.

Table 4 confirms the good reproducibility. The results show that the developed MIPs–leuco fuchsin method can accurately quantify formaldehyde at food-safety-relevant concentrations. The slightly higher recovery may be due to the molecularly imprinted polymer's high affinity and selectivity. Well-defined recognition sites, together with the silica-supported polymer structure, likely enhance the adsorption efficiency and minimize analyte loss during adsorption and desorption. Similar behavior was reported in previous MIP-based systems, where strong template–polymer interactions caused slightly elevated recoveries without compromising method validity.^52^

Formaldehyde was detected in spiked fish samples (Table 4). The clear distinction between blank samples and the fortified control confirms that the observed recovery value reflects accuracy rather than matrix interference or nonspecific colorimetric reactions. The recovery study confirms the method's acceptable accuracy for detecting formaldehyde in fish. However, as accuracy was assessed using fortified concentration, further validation at additional concentration levels is needed to fully establish the method's robustness. Despite this limitation, the results support the MIPs–leuco fuchsin system as a reliable and selective approach for rapid formaldehyde screening of food matrices.

The limit of detection (LOD) can be calculated as 3.3 × (standard deviation/slope). The LOQ is defined as the lowest concentration of an analyte in a sample that can be determined with acceptable precision and accuracy under the stated operational conditions of the method (10 × (deviation standard/slope)). The LOD was 0.152 mg L^−1^, while the LOQ was 0.461 mg L^−1^ (Table 5). A comparative study of the detection limit with the previous methods for the analysis of formaldehyde in food is shown in Table 6.

**Table 5:** Linear regression analysis

Standard solution (ppm)	Absorbance of standard	Absorbance (blank)	Blank concentration (ppm)
0.07	0.008	0.0068	0.029
0.25	0.011	0.0069	0.029
0.5	0.014	0.0062	0.027
0.75	0.018	0.0097	0.098
1	0.020	0.0067	0.029
2	0.029	0.0063	0.027
4	0.048	0.0067	0.029
6	0.071	0.0067	0.028
8	0.090		
10	0.109		
LOD = 3.3 (SD/Slope)	LOD = 3.3 (4.6981 × 10^−4^/0.0102) = 0.152 ppm	Blank absorbance average = 0.007	Blank average concentration = 0.098
LOQ = 10(SD/S)	LOQ = 10 (4.6981 × 10^−4^/0.0102) = 0.461 ppm	SD = 4.6981 × 10^−4^	

**Table 6:** Comparison of methods used for formaldehyde analysis in food

Method	Matrix	LOD	Analysis time (min)	Literature
HPLC	Flour	0.3 ng L^−1^	60	6
GC/MS	Milk	12.20 ng L^−1^	60	8
	Beef	7.8 ng L^−1^		
Colorimetric-spectrometer UV-Vis	Octopus and chicken flesh	30 nM	30	53
Microwell plate titration	Seafood	0 to 200 mM	20	54
Rapid test kit	Fish	0.5–2.0 mg L^−1^	10	
This study	Fish	0.152 mg L^−1^	60	

Table 6 compares the detection limits of several formaldehyde determination methods in food samples. HPLC and GC/MS offer high sensitivity, with LODs in the ng L^−1^ range, but require sophisticated instruments, complex sample preparation, and trained technicians, making them less suitable for routine analysis. Alternatively, a colorimetric UV-vis method for octopus samples gave a 30 nM LOD, but it remains susceptible to interference. The microwell plate titration method shows a detection limit of 0–200 mM with low sensitivity. Using MIPs–leuco fuchsin achieves a 0.152 ppm (152 µg L^−1^) LOD in fish samples. While chromatographic methods offer superior sensitivity (ng L^−1^), our MIP-based method provides a detection limit sufficient for monitoring formaldehyde in food samples, comparable to conventional rapid test kits (0.5–2 mg L^−1^). Using MIPs as formaldehyde-selective materials can increase the sensitivity of colorimetric methods. The SiO_2_@MIP method allows analysis in the same time as HPLC and GC-MS (60 minutes) for sample preparation and analysis. Although it takes longer than some rapid test kits, this method has much higher selectivity and minimizes false positives. Despite the longer analysis time, this developed method can be used as a cheaper alternative.

### Stability and reusability study

**Fig. 10:**
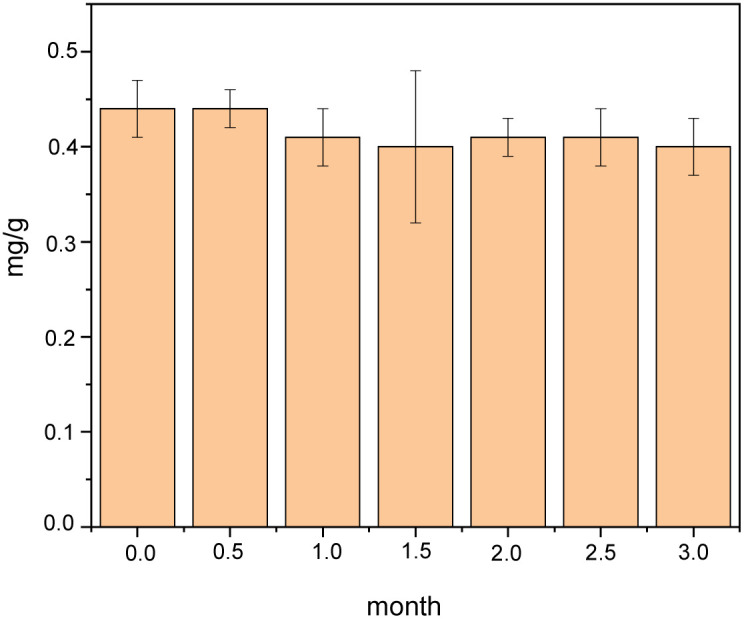
Stability study of SiO_2_@MIPs.

The mechanical properties and stability of SiO_2_@MIPs were studied for practical applications in routine formaldehyde analysis. Notably, after three months of storage at room temperature, SiO_2_@MIPs show a decrease in adsorption capacity of <5% (Fig. 10). The enhanced mechanical stability of this polymer structure is achieved through the silica coating prepared using the Stöber method, which protects the MIPs from degradation and enables reuse in the adsorption–desorption process. Therefore, this method exhibited exceptional long-term stability, maintaining adsorption capacity during storage at room temperature and demonstrating potential for practical applications.

## Conclusions

The mechanical properties and long-term stability of the SiO_2_@MIPs were quantitatively evaluated to assess their suitability for routine formaldehyde analysis. Experimental results indicate that SiO_2_@MIPs maintain high performance over time. After three months of storage at room temperature, the polymer exhibited minimal decrease in adsorption capacity, less than 5%, retaining 0.44 mg g^−1^. This stability is attributed to the silica coating synthesized using the Stöber method, which provides a rigid structural framework and prevents collapse of the imprinted cavities during repeated cycles. The method also demonstrated high reliability in practical applications, with recoveries of between 98.5% and 105% and relative standard deviation (RSD) values ranging from 3.1% to 4.8% (*n* = 3). The limit of detection (LOD) was 0.152 mg L^−1^, which is well below the maximum permissible limits for formaldehyde in food. These quantitative metrics, including high reusability, stable adsorption–desorption efficiency, and long shelf life, confirm that the SiO_2_@MIPs platform is a robust and commercially viable tool for rapid, on-site food safety monitoring. Further testing against structurally similar analogues, such as acetaldehyde and glyoxal, is recommended to validate the method for more complex food matrices.

## Author contributions

Meyliana Wulandari: conceptualization, methodology, and writing – original draft. Muhammad Al Faqih Fiddin and Dieta Putri Ayuserani: data curation and investigation. Rosi Fitri Ramadani: project administration. Syabina Khaila Aliffa: data curation, investigation, and formal analysis. Nofrizal Nofrizal: visualization. Andreas: conceptualization, resources, and methodology. Pandian Bothi Raja: supervision, writing – review & editing. Thomas Nesakumar Jebakumar Immanuel Edison: supervision and validation.

## Conflicts of interest

There are no conflicts to declare.

## Supplementary Material

RA-016-D6RA02680J-s001

## Data Availability

The data supporting this article have been included as part of the supplementary information (SI). Supplementary information is available. See DOI: https://doi.org/10.1039/d6ra02680j.
