# Supplementary material for: Sustainable synthesis of molecularly imprinted polymers using leuco fuchsin for selective detection of formaldehyde in food
Source: RSC Adv. 2026 Jul 8. Online ahead of print. doi: 10.1039/d6ra02680j (PMC13344875; doi:10.1039/d6ra02680j)
Supplement: RA-OLF-D6RA02680J-s001 [file RA-OLF-D6RA02680J-s001.pdf]

Supplementary Information (SI) for RSC Advances.
This journal is © The Royal Society of Chemistry 2026

Supplementary Information

The effect of pH of MIPs and NIP adsorption on target molecules

pH	NIP (mg)	MIPs (mg)
1	0.63478	1.176
2	0.83086	1.225
3	0.73282	1.127
4	0.6838	1.078
5	0.58576	0.931
6	0.48773	0.735
7	0.34067	0.686
8	0.24263	0.637
9	0.29165	0.49
10	0.29165	0.539

The effect of contact time of MIPs and NIP adsorption on target molecules

Time (minutes)	NIP (mg)	MIPs
0	0	0
10	0.147	0.196
20	0.196	0.245
30	0.294	0.392
40	0.343	0.49
50	0.539	0.833
60	0.637	0.882
120	0.735	0.931
180	0.833	1.176
240	0.833	1.225

The effect of optimum concentration of MIPs and NIP adsorption on target molecules

Concentration (ppm)	NIP (mg/g)	MIPs (mg/g)
0	0	0
5	0.1	0.348
10	0.199	0.896
15	2.985	4.478
20	1.99	2.487
25	1.492	1.99
30	1.493	1.99
35	1.492	1.99
40	0.995	1.493
45	0.497	0.995

The equilibrium data for formalin uptake by MIPs and NIP using the Langmuir

1/Ce (NIP)	1/Qe (NIP)	1/Ce (MIPs	1/Qe (MIPs)
0.97101	2.28087	0.97101	7.94328
0.36413	0.89084	0.36413	2.39958
0.30594	0.7584	0.30594	1.59705
0.23592	0.6087	0.23592	1.1807
0.19198	0.49579	0.19198	0.82436
0.17678	0.47239	0.17678	0.73467
0.16184	0.43082	0.16184	0.67001
0.12316	0.34826	0.12316	0.50252
0.07371	0.22331	0.07371	0.33499

The equilibrium data for formalin uptake by MIPs and NIP using the Freundlich model

Log Ce	Log Qe (NIP)	Log Qe (MIPs)
0.43874	-0.38014	0.0502
0.51437	-0.20332	0.1201
0.62724	-0.07214	0.2156
0.71675	0.08388	0.3047
0.75256	0.13391	0.3257
0.79093	0.17392	0.3657
0.90952	0.29884	0.4581
1.13249	0.47497	0.6511

PSA Analyzer

Nonlinear Curve Fit (Gaussian)

Parameters

		Value	Standard Error
Counts	y0	0,14156	0,1878
	xc	111,06483	0,5203
	A	772,90782	25,96036
	w	42,8085	1,34087

Statistics

	Counts
Number of Points	10
Degrees of Freedom	6
Reduced Chi-Sqr	0,18821
Residual Sum of Squares	1,12928
R-Square(COD)	0,99643
Adj. R-Square	0,99464
Fit Status	Succeeded(100)

Summary

	y0		xc
	Value	Standard Error	Value
Counts	0,14156	0,1878	111,06483

ANOVA

		DF	Sum of Squares
Counts	Regression	4	474,87072
	Residual	6	1,12928
	Uncorrected Total	10	476
	Corrected Total	9	316

Fitted Curves Plot

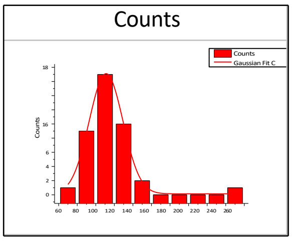

Residual vs. Independent Plot

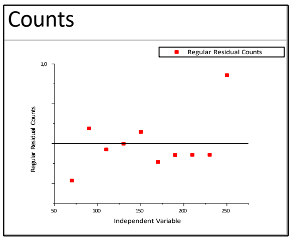

	A		w		Statistics	
Error	Value	Standard Error	Value	Standard Error	Reduced Chi-Sqr	Adj. R-Square
5203	772,90782	25,96036	42,8085	1,34087	0,18821	0,99464

Square	F Value	Prob>F
8,71768	630,76041	6,8993E-8
0,18821		

Parameters

		Value	Standard Error
Counts	y0	0,32158	0,34723
	xc	94,15346	0,61171
	A	758,68349	30,06063
	w	23,63587	1,28306

Statistics

	Counts
Number of Points	6
Degrees of Freedom	2
Reduced Chi-Sqr	0,35143
Residual Sum of Squares	0,70286
R-Square(COD)	0,99884
Adj. R-Square	0,99709
Fit Status	Succeeded(100)

Summary

	y0		xc
	Value	Standard Error	Value
Counts	0,32158	0,34723	94,15346

ANOVA

		DF	Sum of Squares
Counts	Regression	4	869,29714
	Residual	2	0,70286
	Uncorrected Total	6	870
	Corrected Total	5	603,33333

Fitted Curves Plot

Counts

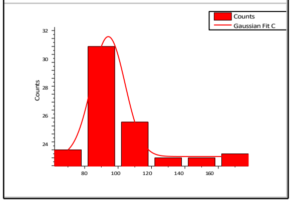

Residual vs. Independent Plot

Counts

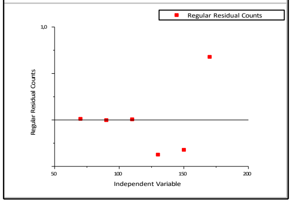

A		w		Statistics	
Value	Standard Error	Value	Standard Error	Reduced Chi-Sqr	Adj. R-Square
758,68349	30,06063	23,63587	1,28306	0,35143	0,99709

Square	F Value	Prob>F
7,32429	618,40341	0,00161
0,35143		

Nonlinear Curve Fit (Gaussian)

Parameters

		Value	Standard Error
Counts	y0	1,39144	0,35492
	xc	88,37125	0,25032
	A	316,56331	16,9676
	w	15,73248	0,91858

Statistics

	Counts
Number of Points	6
Degrees of Freedom	2
Reduced Chi-Sqr	0,27372
Residual Sum of Squares	0,54744
R-Square(COD)	0,99782
Adj. R-Square	0,99455
Fit Status	Succeeded(100)

Summary

	y0		xc
	Value	Standard Error	Value

ANOVA

		DF	Sum of Squares
Counts	Regression	4	517,45256
	Residual	2	0,54744
	Uncorrected Total	6	518
	Corrected Total	5	251,33333

Fitted Curves Plot

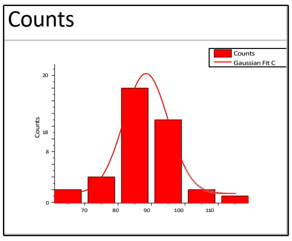

Residual vs. Independent Plot

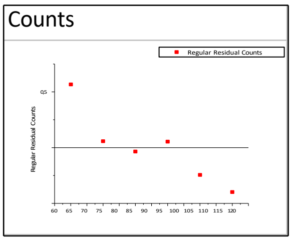

A		w		Statistics	
Value	Standard Error	Value	Standard Error	Reduced Chi-Sqr	Adj. R-Square
316,56331	16,9676	15,73248	0,91858	0,27372	0,99455

Square	F Value	Prob>F
9,36314	472,61309	0,00211
0,27372		
